# Supplementary material for: Cecelia: a multifunctional image analysis toolbox for decoding spatial cellular interactions and behaviour
Source: Nat Commun. 2025 Feb 24;16:1931. doi: 10.1038/s41467-025-57193-y (PMC11850795; doi:10.1038/s41467-025-57193-y)
Supplement: Supplementary file 1 — Supplementary Information [file 41467_2025_57193_MOESM1_ESM.pdf]

Supplementary Information

Cecelia: a multifunctional image analysis toolbox for decoding spatial cellular interactions and behaviour

Dominik Schienstock[1], Jyh Liang Hor[1 ǂ], Sapna Devi[1] and Scott N. Mueller[1*]

[1]Department of Microbiology and Immunology, The University of Melbourne, The Peter Doherty Institute for Infection and Immunity, Melbourne, Australia
[ǂ]Present address: Lymphocyte Biology Section, Laboratory of Immune System Biology, NIAID, NIH, Bethesda, MD, USA

[*] Correspondence: e-mail: smue@unimelb.edu.au

Contents:

Supplementary Figures 1-3

Supplementary Tables 1-2

Supplementary Figure 1

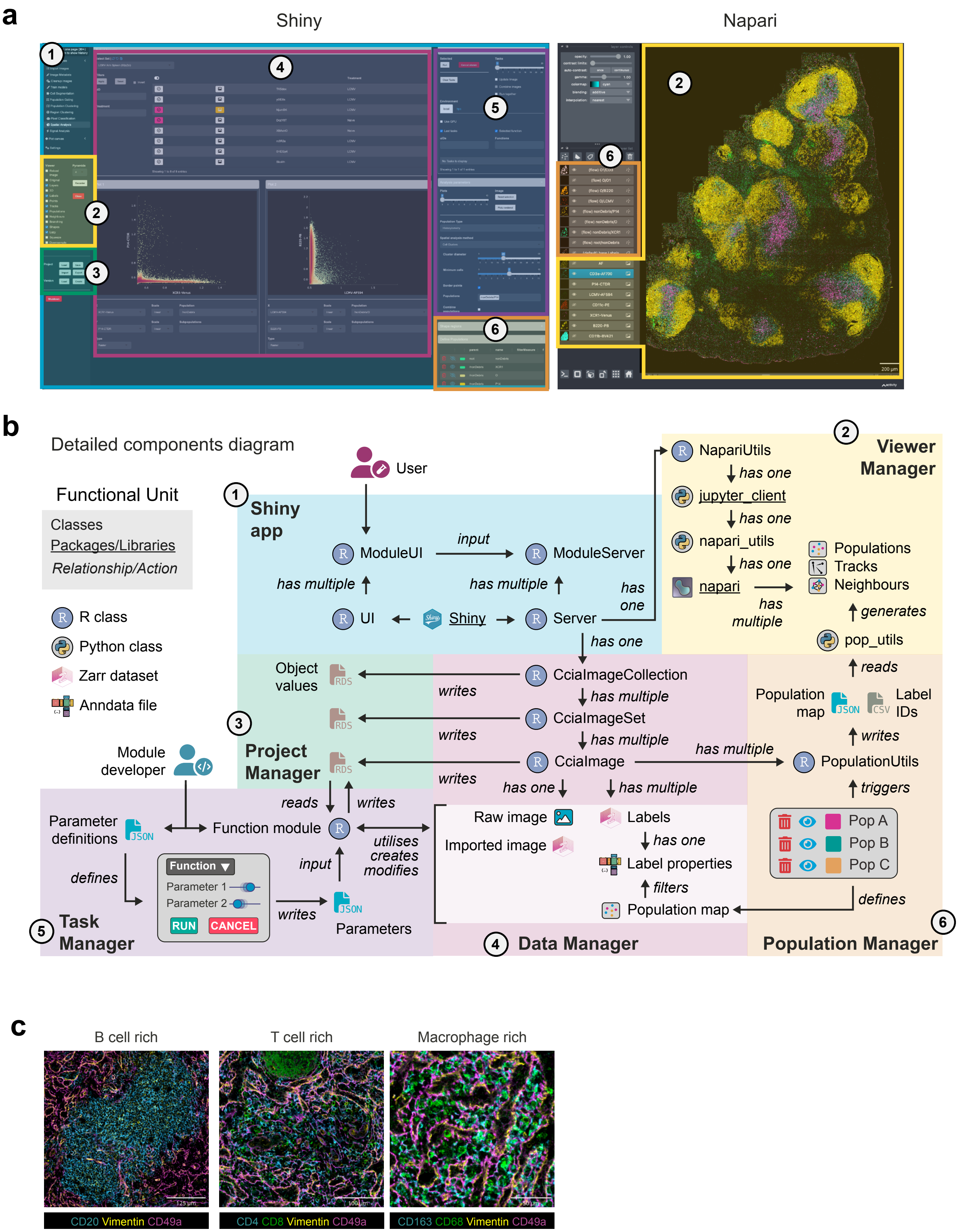

Supplementary Figure 1: Main components of Cecelia

a User interface and **b** the respective underlying software components. (1) The user interacts with the shiny app which is a modular construct of UI and Server components. The main shiny server has one connection to control napari (via NapariUtils) and access data (via CciaImageCollection). (2) Napari is controlled via a jupyter client and a custom napari utilities module. In this way populations, tracks, neighbours, etc. can be toggled from R via Python. (3) The project management class triggers read and write processes of the individual image analysis and the main project values. (4) Each image record has the original raw image file as well as modified versions that the user generates. Each image also contains quantification and further analysis executed by the user. (5) The module developer can provide functions in R and Python to server user requirements. The parameters for each function module can be exposed to user input via structured JSON files. (6) Populations can be generated via different means. The main route is via the PopulationUtils class which holds the population information and can write out structured JSON files and labels identifiers that can be read and displayed by napari. This figure has been designed using resources from Flaticon.com and Fontawesome.com. **c** Examples of cell regions with their respective stromal cell networks from human spleen stained using the IBEX method.

Supplementary Figure 2

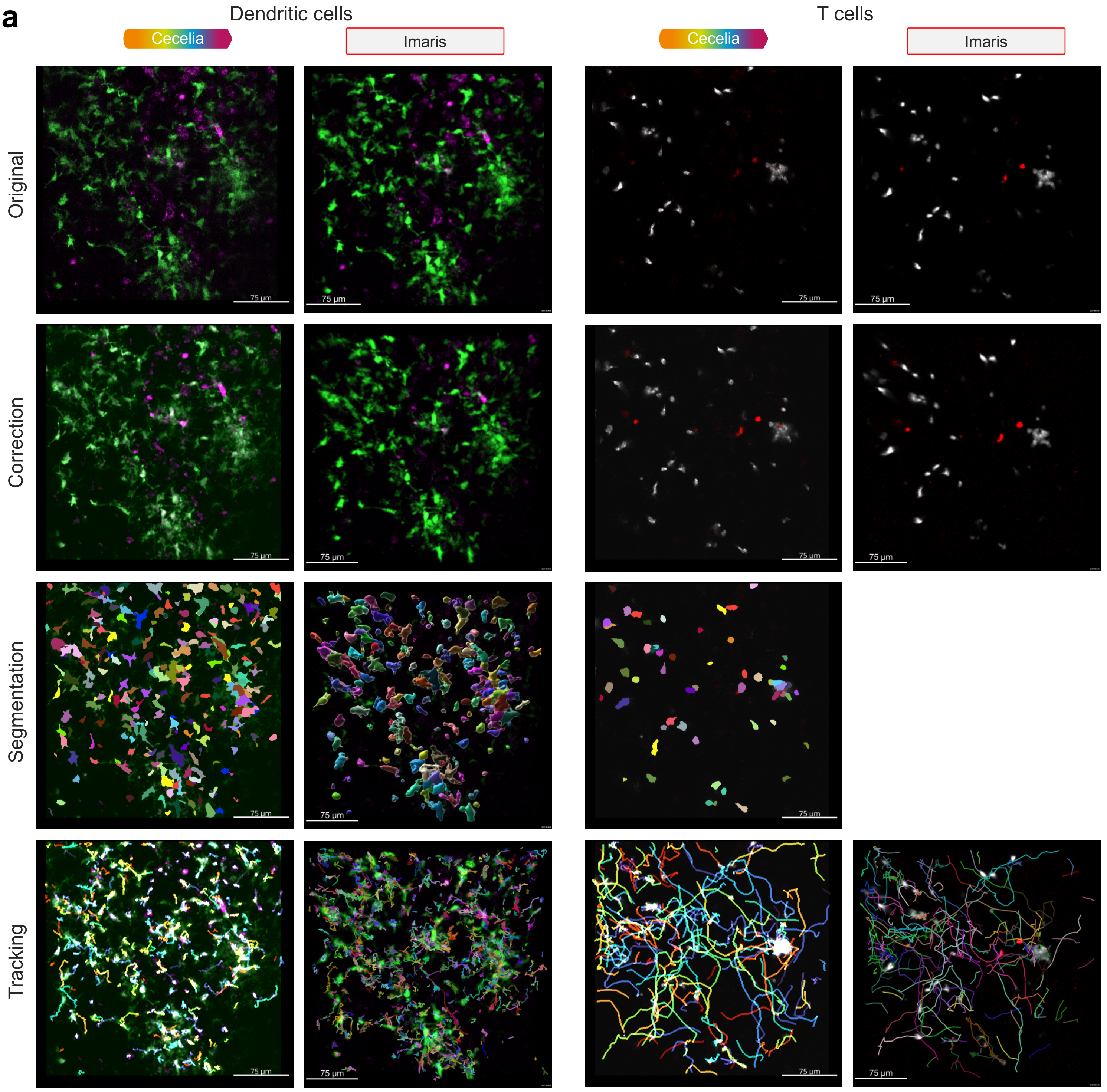

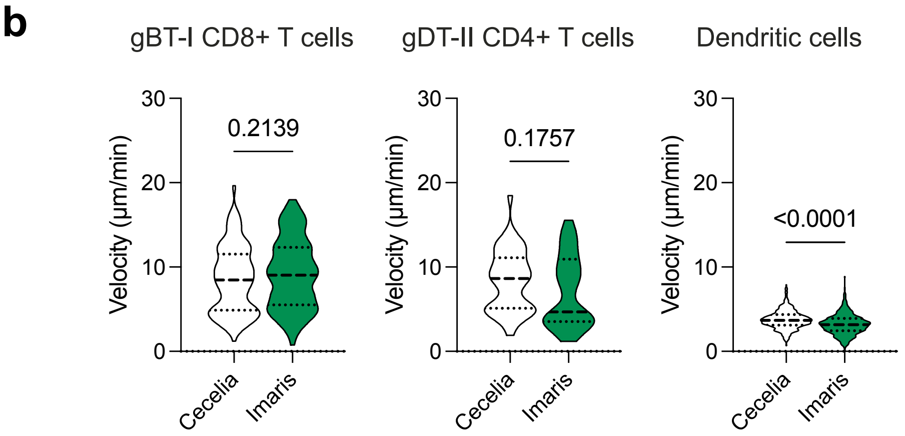

Supplementary Figure 2: Comparison of Cecelia and Imaris to process intravital imaging data

a Two-photon images were processed in similar manner in Cecelia and Imaris. In Cecelia, images were corrected with Cellpose denoising to homogenise the signal intensities within each z-plane. Channels were divided by each other to remove auto-fluorescent artifacts and drift corrected using phase cross-correlation. The corrected channels were subjected to Cellpose segmentation and tracking via btrack. In Imaris, channels were subtracted from each other to remove auto-fluorescent artifacts, surfaces generated using standard watershed processing and tracked using autoregressive motion. The image was drift corrected to a reference frame using these tracking results. **b** Comparison of velocity measurements for T cells and dendritic cells obtained by Cecelia and Imaris (n = 1 image, two-sided t-test).

Supplementary Figure 3

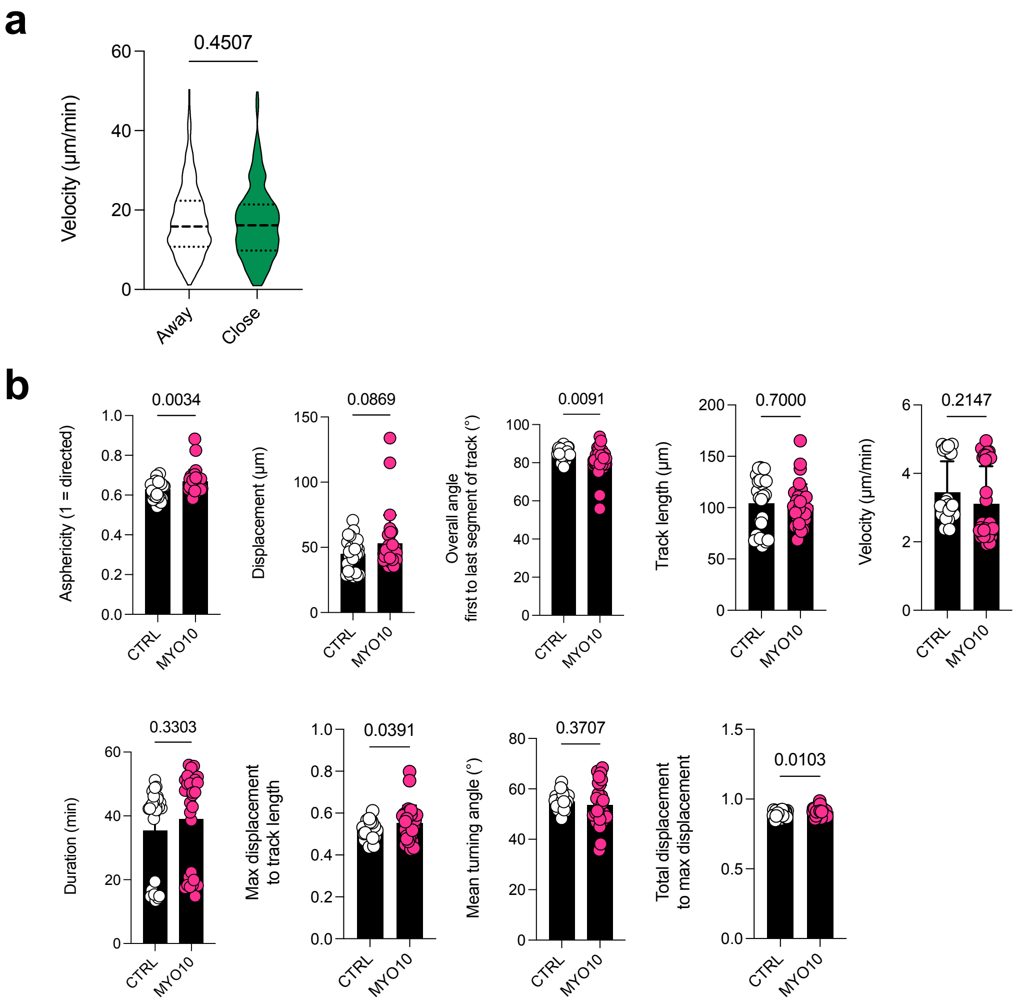

Supplementary Figure 3: Conventional tracking analysis for Figure 5

a Comparative analysis of cell velocity to Fig. 5a with a manual distance threshold to define cells that are close to (≤ 10 µm) and away from (> 10 µm) lymphatics (n = 4, two-sided t-test). **b** Standard track measurements of the data analysed in Fig. 5b (n = 29-30 movies per group from 3 independent experiments, two-sided t-test, mean values +/- SD). Due to technical issues that the authors mention in their publication some of the measurements show a bimodal distribution. For detailed explanation of the individual parameters, we refer to the celltrackR documentation (https://www.rdocumentation.org/packages/celltrackR/versions/1.2.0/topics/TrackMeasures).

Supplementary Table 1: Main packages used in the Cecelia framework

Usage	Package	Language
Data storage	anndata	Python
Tracking of labels	btrack	Python
Cell segmentation and image denoising	cellpose	Python
Tracking measurements	celltrackR	R
Concavity for cell areas	concaveman	R
Viewing images in napari	dask	Python
Cell clusters and contact	dbscan	R
Hidden Markov Models	depmixS4	R
Gating of cell populations	flowWorkspace	R
Bridge between R and Python	jupyter	Python
Population clustering	leidenalg	Python
Image denoising	n2v	Python
Viewing images	napari	Python
Image metadata	ome-types	Python
Managing processes	parallel	R
Interactive plotting and cell population gating	plotly	R
Population clustering	scanpy	Python
General image processing	scikit-image	Python
Data management and processing	Shiny	R
Network branch extraction	skan	Python
Spatial statistics, for example population distributions	spatstat	R
Neighbour analysis	squidpy	Python
General working with image loading/saving	tifffile	Python
3D mesh generation, shapes and contact detection	trimesh	Python
Imaging data	zarr	Python

Supplementary Table 2: Datasets used for figures

Figure	Description	Reference
1d	Confocal mouse spleen	This paper
	CODEX Human cutaneous lymphoma	[1]
	Two-photon Mouse lymph node	[2]
	Two-photon Mouse mammary fatpad	This paper
	IBEX Human spleen	zenodo.org/records/4632320
2	Confocal Mouse spleen LCMV infection	This paper
3a	IBEX Human spleen	zenodo.org/records/4632320
3b	CODEX Human lymph node	portal.hubmapconsortium.org
3c	Xenium Human breast cancer	10xgenomics.com/products/xenium-in-situ/human-breast-dataset-explorer
4a-g	Two-photon Lymph node HSV infection	[2]
4h-k	Two-photon Lymph node HSV infection	This paper
5a	Two-photon Skin DTH-inflamed	app.immunemap.org/experiment-public-view?id=49
5b	Spinning-disk confocal Cancer cell line tissue culture	zenodo.org/records/10539020
S1c	IBEX Human spleen	zenodo.org/records/4632320

1. Phillips, D. et al. Immune cell topography predicts response to PD-1 blockade in cutaneous T cell lymphoma. *Nat Commun* **12**, 6726 (2021).
2. Hor, J.L. et al. Spatiotemporally Distinct Interactions with Dendritic Cell Subsets Facilitates CD4+ and CD8+ T Cell Activation to Localized Viral Infection. *Immunity* **43**, 554-565 (2015).
