# Supplementary material for: Cecelia: a multifunctional image analysis toolbox for decoding spatial cellular interactions and behaviour
Source: Nat Commun. 2025 Feb 24;16:1931. doi: 10.1038/s41467-025-57193-y (PMC11850795; doi:10.1038/s41467-025-57193-y)
Supplement: Supplementary file 2 — Description of Additional Supplementary Files [file 41467_2025_57193_MOESM2_ESM.pdf]

Description of Additional Supplementary Files

Supplementary Movie 1.mp4

Description:

Behavioural analysis of $CD8^+$ and $CD4^+$ T cell responses in lymph nodes draining the site of skin Herpes simplex virus infection. gDT-II $CD4^+$ (cyan) and gBT-I $CD8^+$ (grey) T cells in inguinal lymph nodes 1 day after HSV infection. Cell tracks and spots are coloured by behaviour: directed (red), meandering (white), scanning (blue), aggregating (yellow).
