# Supplementary material for: Cecelia: a multifunctional image analysis toolbox for decoding spatial cellular interactions and behaviour
Source: Nat Commun. 2025 Feb 24;16:1931. doi: 10.1038/s41467-025-57193-y (PMC11850795; doi:10.1038/s41467-025-57193-y)
Supplement: Supplementary file 3 — Reporting Summary [file 41467_2025_57193_MOESM3_ESM.pdf]

nature portfolio

Corresponding author(s): Scott Mueller

Last updated by author(s): Jan 28, 2025

Reporting Summary

Nature Portfolio wishes to improve the reproducibility of the work that we publish. This form provides structure for consistency and transparency in reporting. For further information on Nature Portfolio policies, see our Editorial Policies and the Editorial Policy Checklist.

Statistics

For all statistical analyses, confirm that the following items are present in the figure legend, table legend, main text, or Methods section.

n/a	Confirmed	
☐	☒	The exact sample size (*n*) for each experimental group/condition, given as a discrete number and unit of measurement
☐	☒	A statement on whether measurements were taken from distinct samples or whether the same sample was measured repeatedly
☐	☒	The statistical test(s) used AND whether they are one- or two-sided<br>*Only common tests should be described solely by name; describe more complex techniques in the Methods section.*
☒	☐	A description of all covariates tested
☒	☐	A description of any assumptions or corrections, such as tests of normality and adjustment for multiple comparisons
☐	☒	A full description of the statistical parameters including central tendency (e.g. means) or other basic estimates (e.g. regression coefficient) AND variation (e.g. standard deviation) or associated estimates of uncertainty (e.g. confidence intervals)
☐	☒	For null hypothesis testing, the test statistic (e.g. *F*, *t*, *r*) with confidence intervals, effect sizes, degrees of freedom and *P* value noted<br>*Give P values as exact values whenever suitable.*
☒	☐	For Bayesian analysis, information on the choice of priors and Markov chain Monte Carlo settings
☒	☐	For hierarchical and complex designs, identification of the appropriate level for tests and full reporting of outcomes
☒	☐	Estimates of effect sizes (e.g. Cohen's *d*, Pearson's *r*), indicating how they were calculated

Our web collection on statistics for biologists contains articles on many of the points above.

Software and code

Policy information about availability of computer code

Data collection	Zeiss Zen 2012 was used to collect confocal images. FV30S-SW was used to collect two-photon intravital imaging data.
Data analysis	The presented R-package was develop in RStudio 2023.12.0+369 and R 4.3.0 on MacOS 12.7.6 and is available on github: https://github.com/schienstockd/cecelia. Among the main packages utilised in this framework are the following R and python packages:<br>R: celltrackR (1.10), concaveman (1.10), dbscan (1.1-11), depmixS4 (1.5-0), flowWorkspace (4.12.0), parallel (4.3.0), plotly (4.10.4), shiny (1.8.0), spatstat (3.0.-5)<br>Python: anndata (0.10.8), btrack (0.6.5), cellpose (3.0.10), dask (2024.8.0), jupyter (1.0.0), leidenalg (0.10.2), n2v (0.1.3), napari (0.5.2), ome-types (0.5.1.post1), scanpy (1.10.2), scikit-image (0.24.0), skan (0.11.1), squidpy (1.6.0), tifffile (2024.8.10), trimesh (3.23.5), zarr (2.18.2)

For manuscripts utilizing custom algorithms or software that are central to the research but not yet described in published literature, software must be made available to editors and reviewers. We strongly encourage code deposition in a community repository (e.g. GitHub). See the Nature Portfolio guidelines for submitting code & software for further information.

Data

Policy information about availability of data

All manuscripts must include a data availability statement. This statement should provide the following information, where applicable:

- Accession codes, unique identifiers, or web links for publicly available datasets
- A description of any restrictions on data availability
- For clinical datasets or third party data, please ensure that the statement adheres to our policy

The imaging data generated in this study have been deposited in the Zenodo database under accession code https://doi.org/10.5281/zenodo.14759798. Images not generated in this article were downloaded from the sources outlined in the Supplementary Table 2. Source data are provided with this paper.

Research involving human participants, their data, or biological material

Policy information about studies with human participants or human data. See also policy information about sex, gender (identity/presentation), and sexual orientation and race, ethnicity and racism.

Reporting on sex and gender	Not applicable.
Reporting on race, ethnicity, or other socially relevant groupings	Not applicable.
Population characteristics	Not applicable.
Recruitment	Not applicable.
Ethics oversight	Not applicable.

Note that full information on the approval of the study protocol must also be provided in the manuscript.

Field-specific reporting

Please select the one below that is the best fit for your research. If you are not sure, read the appropriate sections before making your selection.

[x] Life sciences [ ] Behavioural & social sciences [ ] Ecological, evolutionary & environmental sciences

For a reference copy of the document with all sections, see nature.com/documents/nr-reporting-summary-flat.pdf

Life sciences study design

All studies must disclose on these points even when the disclosure is negative.

Sample size	No statistical method was used to determine sample sizes. For intravital imaging, at least 2-3 movies were taken from each mouse in different locations of the tissue. Images from at least 4-5 mice were used for microscopy studies. For public datasets, we aimed to include studies with at least 4 images.
Data exclusions	The decision to exclude images for data analysis was based on their applicability for downstream processing. For intravital imaging, movies with too much noise, autofluorescence or tissue drift were excluded. For confocal imaging, tissues with too much physical damage such as holes or bubbles were excluded. For public datasets, we included all images where possible.
Replication	For data generated in this study, experimental procedures were repeated at least twice on different days with different mice. For public datasets, we aimed to include studies with multiple images where possible to confirm our developed analysis workflows. All datasets from the same source were processed in the same way where possible.
Randomization	For data generated in this study, mice were used between ages 6-12 weeks and randomly allocated to different treatment groups. For public datasets, there was no randomisation in terms of data processing.
Blinding	Blinding was not possible in this study as mice were treated or untreated and injected with labelled cell types. During processing, every image was given metadata to identify treatments, cell types and tissue origin.

Reporting for specific materials, systems and methods

We require information from authors about some types of materials, experimental systems and methods used in many studies. Here, indicate whether each material, system or method listed is relevant to your study. If you are not sure if a list item applies to your research, read the appropriate section before selecting a response.

Materials & experimental systems

n/a	Involved in the study	
☐	☒	Antibodies
☒	☐	Eukaryotic cell lines
☒	☐	Palaeontology and archaeology
☐	☒	Animals and other organisms
☒	☐	Clinical data
☒	☐	Dual use research of concern
☒	☐	Plants

Methods

n/a	Involved in the study	
☒	☐	ChIP-seq
☒	☐	Flow cytometry
☒	☐	MRI-based neuroimaging

Antibodies

Antibodies used	B220 (Pacific Blue, RA3-6B2, BioLegend, Cat: 103227, 1:200), CD3e (AF 700, eBio500A2, Thermo Fischer Scientific, Cat: 56-0033-82, 1:100), LCMV NP (AF 594, VL-4, Bio X Cell, Cat: BE0106, 1:200), CD11b (BV 421, M1/70, BioLegend, Cat: 101251, 1:200)
Validation	Primary antibodies were tested in single stain controls and visually validated for their tissue staining pattern.

Animals and other research organisms

Policy information about studies involving animals; ARRIVE guidelines recommended for reporting animal research, and Sex and Gender in Research

Laboratory animals	C57BL/6, gBT-I, gBT-I.uGFP, OT-I, OT-I.uGFP, P14, P14.ubTomato and XCR1-venus mice were bred in the Department of Microbiology and Immunology, The University of Melbourne. gBT-I mice encode transgenes expressing a T cell receptor recognizing the HSV-1 glycoprotein B-derived epitope gB498-505. OT-I mice encode transgenes expressing a T cell receptor recognizing OVA257-264 peptide. P14 mice encode transgenes expressing a T cell receptor recognizing the LCMV glycoprotein33-41 peptide. Mice were used between ages 6-12 weeks.
Wild animals	Not applicable.
Reporting on sex	All used animals were female. No sex-based analysis was performed.
Field-collected samples	Not applicable.
Ethics oversight	Animal experiments were approved by The University of Melbourne Animal Ethics Committee.

Note that full information on the approval of the study protocol must also be provided in the manuscript.

Plants

Seed stocks	Not applicable.
Novel plant genotypes	Not applicable.
Authentication	Not applicable.
